# Improved Neurological Outcome by Intramuscular Injection of Human Amniotic Fluid Derived Stem Cells in a Muscle Denervation Model

**DOI:** 10.1371/journal.pone.0124624

**Published:** 2015-05-06

**Authors:** Chun-Jung Chen, Fu-Chou Cheng, Hong-Lin Su, Meei-Ling Sheu, Zong-Han Lu, Chien-Yi Chiang, Dar-Yu Yang, Jason Sheehan, Hung-Chuan Pan

**Affiliations:** 1 Department of Medical Research, Taichung Veterans General Hospital, Taichung, Taiwan; 2 Institute of Life Sciences, National Chung-Hsing University, Taichung, Taiwan; 3 Institute of Biomedical Sciences, National Chung-Hsing University, Taichung, Taiwan; 4 Department of Neurosurgery, Chang Bing Show Chwan Memorial Hospital, Changhua, Taiwan; 5 Department of Neurosurgery, University of Virginia, Charlottesville, VA, United States of America; 6 Department of Neurosurgery, Taichung Veterans General Hospital, Taichung, Taiwan; 7 Faculty of Medicine, School of Medicine, National Yang-Ming University, Taipei, Taiwan; University of Minnesota Medical School, UNITED STATES

## Abstract

**Purpose:**

The skeletal muscle develops various degrees of atrophy and metabolic dysfunction following nerve injury. Neurotrophic factors are essential for muscle regeneration. Human amniotic fluid derived stem cells (AFS) have the potential to secrete various neurotrophic factors necessary for nerve regeneration. In the present study, we assess the outcome of neurological function by intramuscular injection of AFS in a muscle denervation and nerve anastomosis model.

**Materials and Methods:**

Seventy two Sprague-Dawley rats weighing 200–250 gm were enrolled in this study. Muscle denervation model was conducted by transverse resection of a sciatic nerve with the proximal end sutured into the gluteal muscle. The nerve anastomosis model was performed by transverse resection of the sciatic nerve followed by four stitches reconnection. These animals were allocated to three groups: control, electrical muscle stimulation, and AFS groups.

**Results:**

NT-3 (Neurotrophin 3), BDNF (Brain derived neurotrophic factor), CNTF (Ciliary neurotrophic factor), and GDNF (Glia cell line derived neurotrophic factor) were highly expressed in AFS cells and supernatant of culture medium. Intra-muscular injection of AFS exerted significant expression of several neurotrophic factors over the distal end of nerve and denervated muscle. AFS caused high expression of Bcl-2 in denervated muscle with a reciprocal decrease of Bad and Bax. AFS preserved the muscle morphology with high expression of desmin and acetylcholine receptors. Up to two months, AFS produced significant improvement in electrophysiological study and neurological functions such as SFI (sciatic nerve function index) and Catwalk gait analysis. There was also significant preservation of the number of anterior horn cells and increased nerve myelination as well as muscle morphology.

**Conclusion:**

Intramuscular injection of AFS can protect muscle apoptosis and likely does so through the secretion of various neurotrophic factors. This protection furthermore improves the nerve regeneration in a long term nerve anastomosis model.

## Introduction

Peripheral nerve injuries result in degeneration of nerve fibers and denervation of the innervated muscle. Following injury to nerve supplying skeletal muscles, the effected organ (i.e. the skeletal muscle) not only develops various degree of morphological changes such as atrophy, but it also results in qualitative changes such as reduced contractile and metabolic function. Cell apoptosis plays a critical role in denervated muscle atrophy and degeneration [1,2]. Reducing or postponing cell apoptosis could provide treatment for skeletal muscle atrophy and degeneration [3,4].

Muscle denervation reduces mitochondrial contents and produces muscle atrophy [1]. Skeletal muscle mitochondria exist as a retinaculum that projects from below the sarcolemmal membrane and extend to intermingle within the myofibrils. Cytochrome c and apoptosis-inducing factor are pro-apoptotic factors that can be released from these mitochondria through a specialized channel termed the mitochondrial permeability transition pore (mtPTP), leading to DNA fragments [5–10]. The mtPTP is regulated by Bcl-2 family members, including pro-apoptotic Bax, which facilitates pore opening, and anti-apoptotic Bcl-2, which inhibits pore opening [11–13]. Furthermore, denervated muscle has greater mitochondrial apoptotic susceptibility, which coincided with an increased ratio of Bax to Bcl-2 [6]. Thus, a reduction in apoptosis raises the possibility of lessening muscle atrophy and increasing muscle regeneration.

In general, skeletal muscle fibers may regenerate after an injury through expressing neurotrophic factors which are essential for muscle regeneration [14–16]. For muscle regeneration in spindle formation and myotube, BDNF, NT-3, and CNTF are responsible for this event [17–19]. Thus, neurotrophic factors display a crucial role in denervated muscle regeneration.

Stem cell therapy is a potential promising approach for the treatment of muscular dystrophies such as Duchenne muscular dystrophy, in which muscle fiber degenerates due to lack of the protein dystrophin [20,21]. Skeletal muscle regeneration is mainly mediated by muscle-specific stem cells called satellite cells. Their progeny, myoblast, can be expanded in culture, and myoblasts retain myogenic differentiation capacity [22]. When human mesenchymal stem cells are transplanted into a model of Duchenne muscular dystrophy, the stem cells contributed to myofibers and functional satellite cells, restore sarcolemmal expression of dystrophin, and enhance the expression of neurotrophic factors [23,24]. Thus, mesenchymal stem cell demonstrated high therapeutic potential for restoration of muscle function.

Amniotic fluid-derived stem cells (AFS) have been proposed as candidates for many neurological degenerated diseases [25]. In our laboratory, we have demonstrated that transplantation of AFS might promote sciatic nerve regeneration through the secretion of CNTF and NT-3 [26,27]. Furthermore, AFS and GDNF-transfected AFS transplantation improved outcome by both the modulation of the inflammatory process and increases secretion of neurotrophic factors [28–30]. Thus, the transplantation of AFS may provide the neurotrophic factors essential for the muscle regeneration process.

Given the inherent characteristics of neurotrophic factors secretion in human AFS and the neurotrophic factors essential for muscle regeneration, we investigated the feasibility of AFS transplantations by injection into denervated muscle. We also assessed the effects of AFS transplantation in terms of the nerve and muscle function achieved using both muscle denervation and nerve anastomosis models.

## Material and Methods

Many common essays listed here have been described in several previous works. However, a summary of the methods is detailed below for the sake of completeness and clarity [31].

The animal study either denervated model or nerve anastomosis was conducted in strict accordance with recommendation in the guide for the care and use of laboratory animals of the National Institute of Health. The protocol was approved by the committee on the ethics of animals experiments of Taichung Veterans General Hospital (La-101916). All surgery was performed in animals anesthetized with 4% isoflurane in induction followed by a maintenance dose (1%-2%), and all efforts were made to minimize suffering.

The method for culturing AFS has been described previously [26]. AFS cells were cultured in 5 ml of β-minimum essential medium (β-MEM; Gibco-BRL) supplemented with 20% fetal bovine serum (FBS; Hyclone, Logan, UT, USA) and 4 ng/ml basic fibroblast growth factor (bFGF; R&D system, Minneapolis, MN, USA) in a 25 cm flask and incubated at 37°C with 5% humidified CO_2_. This protocol was approved by the Institutional Review Board of the Veterans General Hospital (950203/C06022).

### Denervated animal models

Thirty-six SD rats weighing 250–300g were used in the denervation model for the current study. The left sciatic nerve approach was conducted under a microscope using the gluteal muscle splitting method. The left sciatic nerve 10 mm from the obturator was transected, and the proximal end of the nerve was sutured to the muscle to avoid the spontaneous regeneration as a denervation model. These animals were randomly assigned to one of three groups including a control group with 100 μl PBS injection over the gastrocnemius muscle (n = 12), percutaneous electrical muscle stimulation (positive control) (ES group) (n = 12), and AFS group (n = 12) with a total of 5x10^6^ of AFS cells diluted in PBS with final volume of 100 μl intramuscularly injected within 30 minutes to the belly of the gastrocnemius muscle along three points using a 26-gauge Hamilton syringe attached to an automated pump and left in situ for an additional 5 minutes to avoid reflux along the injection tract. In the electrical stimulation group, the paradigm featured a treatment consisting of 30 minutes per day using 400 ms of 100 Hz frequency, 200 μs per phase biphasic pulses with 6 seconds of rest (ElePuls HV-F125, Omron, Japan). Stimulation amplitudes were adjusted to maintain a high visual muscle contraction.

All animals were trained and treated in accordance with our previous study [31]. These animals were euthanized 2 weeks after operation for analysis including histomorphological evaluation (n = 6 for each group with a total of 18) and western blot protein analysis (n = 6 for each group with a total of 18).

### Nerve anastomosis

For this portion of the research, thirty-six SD rats weighing 250–300g were anesthetized. The left sciatic nerve was transected 10 mm from the obturator and an anastomosis was performed using a four stitches method. After the nerve anastomosis, the animals were assigned to one of three groups as follows: Group I: nerve anastomosis as a control with 100 μl PBS intramuscular injection (control group) (n = 12); Group II: percutaneous electrical stimulation as a positive control (paradigm the same as denervated model) (ES group) (n = 12); Groups III: AFS with 5x10^6^ cells intramuscularly injected to gastrocnemius muscle and the paradigm was the same with denervated model (AFS group) (n = 12). The animals were allowed to receive neurological assessment such as SFI and CatWalk gait analysis pre-operative and weekly after operation till to the end of the experiment [31]. In addition, all animals were subjected to histomorphological assessment and electrophysiology two months after operation (n = 6 for each group). Six animals from each group for CTB neuronal traction were conducted two months after operation.

### Sciatic function index (SFI) and gait analysis

A technician who was blinded to the assignment of animals to measure sciatic nerve function every week using the sciatic function index (SFI) as described in our previous study [31]. The methodology for conducting the CatWalk gait analysis has been previously described [31]. In brief, the CatWalk XT system comes with a high-speed digital camera with a sample rate of 100 frames per second. The video camera transforms each scene (i.e. the area in front of the lens) into a digital image (i.e. an image composed of discrete pixels of digital brightness values). The digital images are transferred to a computer through an Ethernet connection. The brightness of a pixel depends on the amount of light received from such an area by the camera. The Illuminated Footprint enables intensity difference to be detected between animals’ paws. The 3D footprint intensity tab plots the print intensity of the four paws for each frame in which the paws have contact with the glass plate in a 3D chart. The intensities vary from 0 to 225, and they are represented by different colors. A 3D chart can be rotated in all directions. Quantitative analysis of the data from the Catwalk XT includes the following parameters: step sequence distribution, regularity index (RI), print area, swing and stance phases, and intensity.

### Electrophysiological study

Sciatic nerves from individual groups were exposed 8 weeks after operation, and electrical stimulation was applied to the proximal side of the injured site. The evoked compound muscle action potential (CMAP) amplitudes and conduction latencies were recorded in the gastrocnemius muscle with an active monopolar needle electrode 10 mm below the tibia tubercle and with a reference needle 20 mm from the active electrode. The stimulation intensity and filtration ranges were 5 mA and 20–2000 Hz, respectively. A similar assessment was performed on the non-injured side. The CMAP data and conduction latency were converted to the ratio of the injured side divided by the normal side to adjust for the effect of anesthesia [31].

### Retrograde labeling of spinal motoneurons and dorsal root ganglion neurons

To label spinal motoneurons and dorsal root ganglion neurons, the tracer was then placed at the cut end of sciatic nerve 1cm distal to the anastomosis in the polyethylene capsule containing a solution of 1% cholera toxin subunit B low salt (CTB; List Biological Laboratories, Campbell, CA, USA) and left in place for 30 minutes. The capsule was then removed, and the surrounding area was rinsed with saline. Twenty-four hours later, these animals (6 rats for each group) were deeply re-anesthetized and perfused via the ascending aorta with 200 ml saline at body temperature, followed by 700 ml of ice cold 4% (W/V) paraformaldehyde in a 0.15-M phosphate buffer (pH 7.2–7.4). The L5 spinal cord segment and associated dorsal root ganglion was carefully removed. The L5 spinal cord was post-fixed for 4 hours and cryoprotected in sucrose (30% in 0.01MPBm pH 7.3) at 4°C overnight. Frozen cross sections were cut at 20μm with Cryostat (Mocram HM 560, Heidelberg, Germany) and every fifth section was thaw mounted serially in a rostro-caudal sequence on gelatin coated slide.

### Western blot

The distal end of nerve and middle part of gastrocnemius muscle was harvested two weeks after various treatment and proteins were extracted. Proteins (50 μg) were resolved by SDS-polyacrylamide gel electrophoresis and transferred onto a blotting membrane. Antibodies against Bcl-2 (1:1000, BD), Bad (1:1000, Santa Cruz), Bax (1:1000, Santa Cruz), 8-oxo-dG (1:500, R&D), desmin (1:200, Proteintech), acetylcholine receptor (1:200, Millipore), GAPDH (1: 1000, Santa Cruz) were incubated overnight at 4°C. AFS and fibroblast cell lysated protein (50 μg) was separated by SDS-PAGE electrophoretically transferred to nitrocellulose membranes. After blocking, the blots were incubated with antibodies for NT-3 (1:1000, Millipore), BDNF (1:1000, Abcam), CNTF (1:1000, Abbiotec), GDNF (1:1000, Abbiotec), and GAPDH (1:1000, Santa Cruz). Horseradish peroxidase-conjugated secondary antibodies were used. Protein bands were analyzed by the ISI1000 image system (Alpha Innotech Corporation, CA, USA).

### Caspase activity assay

The middle part of gastrocnemius muscle was harvested 2 weeks after injury and homogenized on ice in a lysis buffer containing 20 mM HEPES, pH 7.4, 4 mM EDTA, 1 mM EGTA, 5 mM MgCl_2_, and 1 mM DTT. An aliquot of 50 μl of the supernatant was incubated with an equal volume of the reaction buffer containing 20 mM HEPES, pH 7.4, 4 mM EDTA, 0.2% CHAPS, 10 mM DTT and flourogenic peptide substrate Ac-DEVD-AMC. Enzymatic release of free AMC was measured at an excitation of 380 nm and an emission of 460 nm. Arbitrary activity was expressed as the fluorescence change per amount of protein. The data was presented as the percentage of various treatment divided by the normal side.

### Enzyme-linked immunosorbent assays (ELISA)

The concentration of NT-3, BDNF, CNTF, GDNF in the cultured supernatant from human AFS and fibroblast cells was measured with enzyme immunoassay kit (R &D). The distal end of nerve and middle part of gastrocnemius muscle tissues were homogenized in buffer containing 1% Triton X-100, 50 mM Tris-HCl, 150 mM NaCl, and 1% protease inhibitor cocktail (Calbiochem). The obtained homogenates and cultured supernatants were loaded onto 96-well plates at 4°C overnight. After washing with 0.1% Tween-20/PBS and blocking, the wells were incubated with indicated primary antibodies and followed by biotin-conjugated second antibody and streptavidin-HRP. Finally, the color was developed by the addition of TMB and the optical density was measured using microplate reader at wavelength 450 nm.

### Immunofluorescence staining of AFS

AFS cells and human fibroblast cells were cultured for 24 hours and then fixing them with 1 ml of 4% paraformaldehyde in PBS and further blocked, reacted overnight at 4°C with anti-human antibody of NT-3 (1:200, Millipore), BDNF (1:200, Abcam), CNTF (1:200, Abbiotec), and GDNF (1:200, Abbiotec). After washing, the slides were incubated for 1 hour at room temperature with anti-rabbit immunoglobulins-FITC and then viewed on a fluorescence microscope.

### Immunohistochemistry

Sciatic nerve and gastrocnemius muscle were subjected to immunohistochemistry with antibodies against NT-3 (1:200, Millipore), BDNF (1:200, Abcam), CNTF (1:200, Abbiotec), GDNF (1:200, Abbiotec), S-100 (1:200, Serotec), neurofilament (1:200, Millipore), desmin (1:200, Proteintech), acetylcholine receptor (1:200, Millipore) and 8-oxo-dG (1:500, R&D) for detection of nerve and muscle regeneration. AF 488 donkey anti-mouse IgG and AF594 donkey anti-rabbit (Invitrogen; 1:200 dilutions) were used to stain and viewed by confocal microscopy capture image. Five consecutive resections (approximately maximum diameter) were chosen and measured by the IS 1000 image analysis system according to our previous study [31]. The number of cells was counted in randomly selected 20 squares from100 squares in an ocular grid. The region of maximum diameter of the resected nerve tissue and muscle was chosen to be examined. Areas of activities (0.2 mm^2^) appeared dense against background and were measured.

### Histology examination

After neurobehavioral and electrophysiological testing, six rats in each group underwent transcardial perfusion with 4% paraformaldehyde in 0.1 M phosphate buffer (pH 7.4) after being re-anesthetized. The bilateral gastrocnemius muscle detached from the bone was sent for measurement of muscle weight. The spinal cord were harvested and cut transversely for assessment of morphology of anterior horn cells. Left sciatic nerve and muscle was harvested from the animals after electrophysiological testing and the nerve tissue was fixed on a plastic plate using stay suturing to keep the nerve straight. The nerve and muscle were embedded, cut longitudinally into sections 8 μm thick and stained with haematoxylin-eosin (H&E) for the measurement of morphology and Luxol fast blue for determination of myelination.

### Statistical analysis

All data are expressed as mean ± SE (standard error). Statistical analysis was performed with SPSS (version 22.0). Differences between study groups were assessed with ANOVA followed by Dunnett’s test or Bonferroni’s multiple comparisons. A *p* value less than 0.05 was considered significant.

## Results

### Intramuscular injection of AFS protects against nerve degeneration and muscle apoptosis

**Fig 1:**
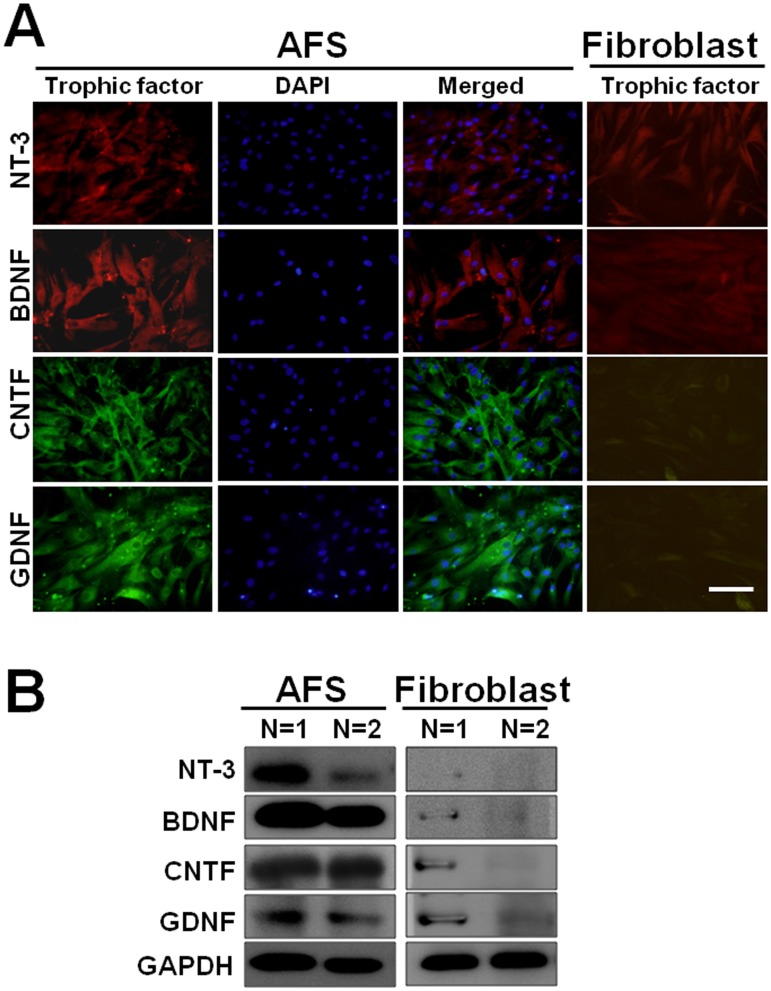
Illustration of neurotrophic factors in AFS. (A) Positive stating of NT-3, BDNF, CNTF, and GDNF in AFS, counter- staining with DAPI and merged imaging; human fibroblast cells as a negative control (B) The demonstration of expression of NT-3, BDNF, CNTF, and GDNF after Western blot analysis in AFS and human fibroblast cells in two different samples. Bar length = 100 μm.

**Fig 2:**
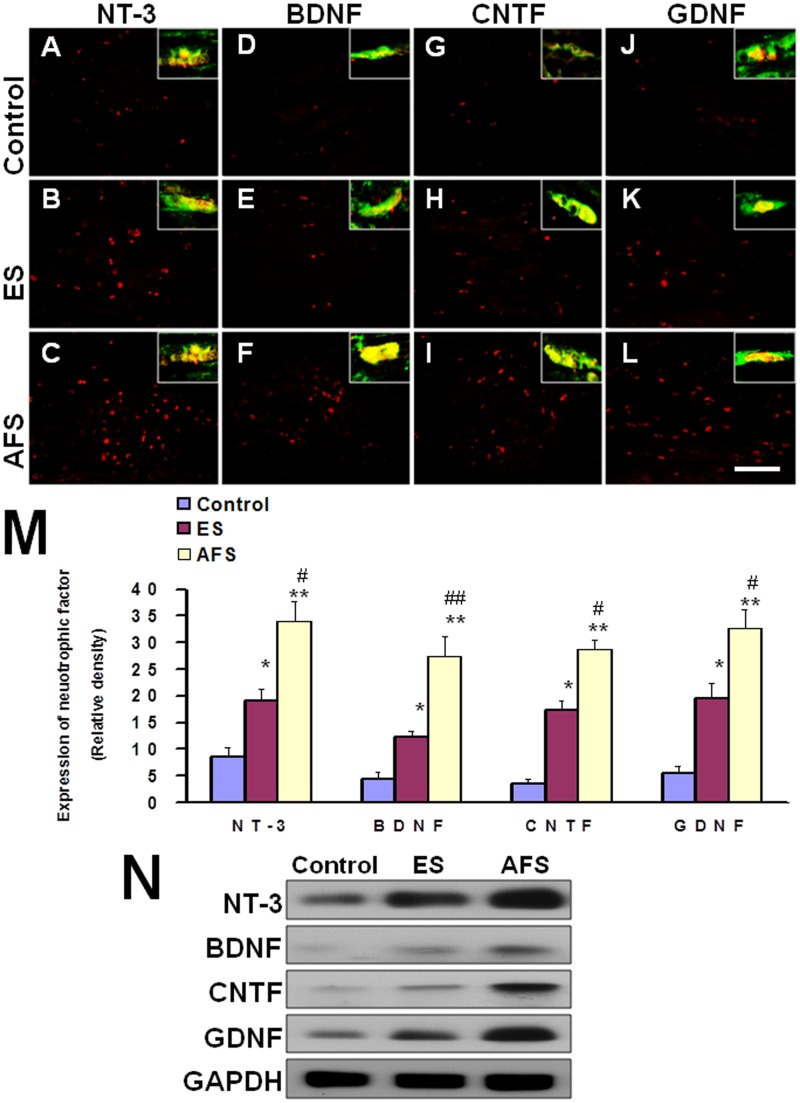
Representative of expression of neurotrophic factors in the distal end of nerve 2 weeks after muscle denervation. (A, B, C) expression of NT-3 in control, ES, and AFS group (D, E, F) expression of BDNF in control, ES, and AFS group (G, H, I) expression of CNTF in control, ES, and AFS group (J, K, L) expression of GDNF in control, ES, and AFS group (M) Quantitative analysis of neurotrophic factors in different treatment group.(N) The representative of western blot analysis in different treatment related to expression of various neurotrophic factors Intersects in each microphotography (yellow) showed the magnification of co-location of neurotrophic factors (red) with S-100 (green) Upper Bar length = 100 μm, N = 6 for each group; Control, ES, and AFS: see text, *p<0.05 and ** p<0.001indicated a significant difference as compared to the control group; # indicated p<0.05 as compared to the ES group.

Human AFS was subjected to immunohistochemistry staining, and positive expression of NT-3, BDNF, CNTF, and GDNF was demonstrated as compared to human fibroblast as a negative control (Fig 1A). The western blot analysis also revealed substantial expression of NT-3, BDNF, CNTF, and GDNF in AFS cells (Fig 1B). The ELISA analysis in Table 1 showed there were significant difference in the supernatant between AFS and fibroblast cell among the various neurotrophic factors. Percutaneous electrical muscle stimulation (ES) as a positive control 2 weeks after muscle denervation showed a statistically different increase of NT-3 (19.1±2.1 vs. 8.6±1.8 relative density; p<0.05) (Fig 2B, 2A and 2M), BDNF (12.2±1.1 vs. 4.5±1.3 relative density; p<0.05) (Fig 2E, 2D and 2M), CNTF (17.3±1,8 vs 3.6±0.8 relative density; p<0.05) (Fig 2H, 2G and 2M), and GDNF (19.5±2.9 vs. 5.5±1.3 relative density, p<0.05) (Fig 2K, 2J and 2M) over the distal end of nerve as compared to the control group. Human AFS injection also increased the expression of NT-3 (34.1±3.6 relative density) (Fig 2C), BDNF (27.4±3.8 relative density) (Fig 2F), CNTF (28.7±1.8 relative density) (Fig 2I), and GDNF(32.4±3.4 relative density) (Fig 2L) either compared to control (p<0.01, p<0.01, 0<0.01, p<0.01, respectively) or ES group (p<0.05, p<0.01, p<0.05, p<0.05, respectively) (Fig 2M). Most of neurotrophic factors expressions were co-localized with staining of S-100 (Fig 2A–2L). The western blot analysis also showed the same trends (Fig 2N). The quantitative analysis by ELISA in Table 2 substantially showed the significant difference in treatment related to the expression of various neurotrophic factors.

**Table 1:** Determination of neurotrophic factors in supernatant from the culture medium in AFS and fibroblast cell culture by ELISA.

	AFS	Fibroblast	P values
NT-3	0.55±0.02 OD	0.33±0.02 OD	P<0.01
BDNF	0.35±0.02 OD	0.21±0.004 OD	P<0.01
CNTF	0.33±0.02 OD	0.063±0.005 OD	P<0.001
GDNF	0.28±0.03 OD	0.094±/0.004 OD	P<0.01

The data was presented as mean±standard error.

The abbreviation of OD, AFS, NT-3, BDNF, CNTF, and GDND: see text.

**Table 2:** Results of neurotrophic factors determined by ELISA in distal end of nerve.

	Control	ES	AFS	P values
NT-3	0.488±0.05 OD	0.697±0.016 OD	0.989±0.026 OD	P<0.001
BDNF	0.36±0.047 OD	0.647± OD	0.789±0.034 OD	P<0.01
CNTF	0.251±0.014 OD	0.372±0.028 OD	0.527±0.03 OD	P<0.001
GDNF	0.274±0.027 OD	0.622±0.04 OD	0.776±0.029 OD	P<0.001

The data was presented as mean±standard error.

The abbreviation of OD, NT-3, BDNF, CNTF, GDNF, control, ES, AFS: see text.

**Fig 3:**
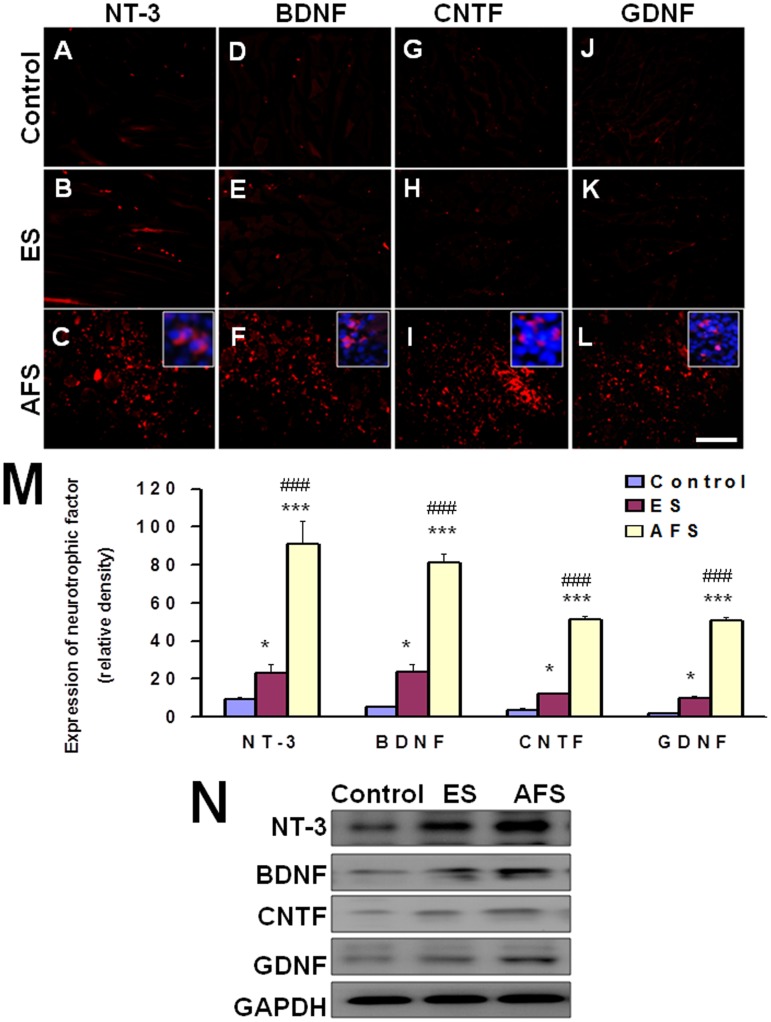
Representative of expression of neurotrophic factor in the denervated muscle 2 weeks after denervation. (A, B, C) expression of NT-3 in control, ES, and AFS group (D, E, F) expression of BDNF in control, ES, and AFS group (G, H, I) expression of CNTF in control, ES, and AFS group (J, K, L) expression of GDNF in control, ES, and AFS group (M) quantitative analysis of neurotrophic factors in different treatment group. (N) The representative of western blot analysis in different treatment related to expression of various neurotrophic factors. Bar length = 100 μm, N = 6, Control, ES, and AFS: see text. Blue color in C, F, I, and L showed the magnification of Hoechst 33342 merged with various neurotrophic factors of NT-3, BDNF, CNTF, and GDNF. Bar length = 100 μm, N = 6 for each group, * p<0.05 and *** p<0.001indicated a significant difference as compared to the control group; ### p<0.001 indicated a significant difference as compared to the ES group.

In denervated muscle, ES caused significant elevation of NT-3 (23.2±4.3 vs. 9.1±1.5 relative density, p<0.05) (Fig 3B, 3A and 3M), BDNF (23.4±4.4 vs. 4.9±0.6 relative density, p<0.05) (Fig 3E, 3D and 3M), CNTF (12.1±0.3 vs. 3.5±0.8 relative density, p<0.05) (Fig 3H, 3G and 3M), and GDNF (9.6±1.1 vs. 1.9±0.3 relative density, p<0.05) (Fig 3K, 3J and 3M) over the gastrocnemius muscle relative to the control group. In the AFS injection group, there were significant elevation of NT-3 (91.1±11.8 relative density) (Fig 3C), BDNF (80.9±4.8 relative density) (Fig 3F), CNTF (51.2±1.7 relative density) (Fig 3I), and GDNF (50.9±1.7 relative density) (Fig 3L) over the gastrocnemius muscle when compared to either the control (p<0.001, p<0.001, p<0.001, p<0.001, respectively) or the ES group (p<0.001, p<0.001, p<0.001, p<0.001, respectively) (Fig 3M). Most of neurotrophic factors in the AFS groups were distributed in the AFS cells as demonstrated by Fig 3 panels C, F, I, and L. The western blot analysis also showed the same trends (Fig 3N). The quantitative analysis by ELISA in Table 3 substantially showed the significant difference in treatment related to the expression of various neurotrophic factors.

**Table 3:** Results of neurotrophic factors determined by ELISA in denervated muscle.

	Control	ES	AFS	P values
NT-3	0.39±0.12 OD	0.566±0.057OD	0.834±0.032OD	P<0.001
BDNF	0.499±0.045 OD	0.678±0.063 OD	1.27±0.067 OD	P<0.001
CNTF	0.274±0.039 OD	0.538±0.074 OD	0.957±0.095 OD	P<0.01
GDNF	0.348±0.164 OD	0.704±0.041 OD	0.867±0.058 OD	P<0.05

The data was presented as mean±standard error.

The abbreviation of OD, NT-3, BDNF, CNTF, GDNF, control, ES, AFS: see text.

**Fig 4:**
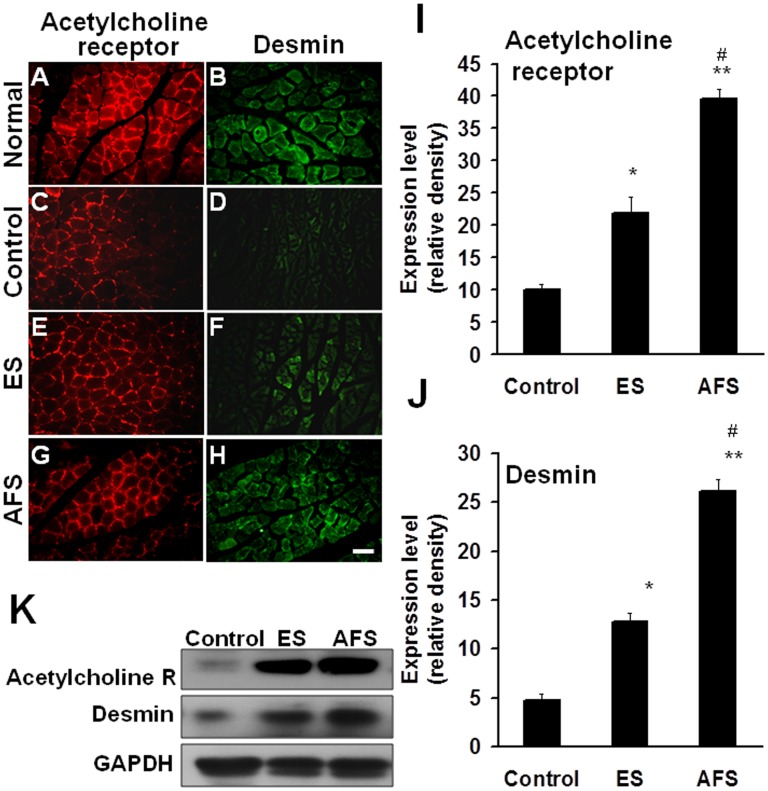
Representative of acetylcholine receptor and desmin two weeks after muscle denervation. (A, C, E,G) Expression of the density of acetylcholine receptor in normal muscle and the denervated muscle subjected to treatment of control, ES, and AFS groups.(B, D, F, H) Expression of the density of desmin in normal muscle and the denervated muscle subjected to treatment of control, ES, and AFS groups (I) Quantitative analysis of the acetylcholine receptor in different treatment groups (J) Quantitative analysis of desmin in different treatment groups. (K) The representative of western blot analysis in different treatment related to expression of desmin and acetylcholine R (receptors). Bar length = 100 μm, N = 6, * p<0.05 and *** p<0.001indicated a significant difference as compared to the control group; ### p<0.001indicated a significant difference as compared to the ES group.

Two weeks after muscle denervation, the ES group showed significant preservation of muscle function and morphology such as the acetylcholine receptor (21.9±2.5 vs. 10.1 ±0.8 relative density, p<0.05) (Fig 4E, 4C and 4I) and desmin (12.8±0.8 vs. 4.7±0.7 relative density, p<0.05) (Fig 4F, 4D and 4J) as compared to the control group. In the AFS injection group, there were significantly increased expression of the acetylcholine receptor (39.6±1.4 relative density) (Fig 4G, 4E, 4C and 4I), and desmin (26.1±1.2 relative density) (Fig 4H, 4F, 4D and 4J) compared to either the control (p<0.01, p<0.01, respectively) or the ES group (p<0.05, p<0.05, respectively) (Fig 4I and 4J). The morphology of denervated muscle after AFS injection reached those at the normal group either in acetylcholine receptor and desmin (Fig 4A, 4B, 4G and 4H). The western blot analysis also showed the same trends (Fig 4K). The quantitative analysis by ELISA in Table 4 substantially showed the significant difference in treatment related to the expression of desmin and acetylcholine receptors.

**Table 4:** Results of Acetylcholine receptor and desmin in denervated muscle related to different treatment and time frame.

	Control	ES	AFS	P values
Acetylcholine R (2weeks)	0.746±0.11 OD	1.163±0.137 OD	1.488±0.14 OD	P<0.05
Acetylcholine R (2 months)	0.688±0.101 OD	1.309±0.028 OD	1.521±0.055 OD	P<0.001
Desmin (2 weeks)	0.169±0.034 OD	0.386±0.02 OD	0.749±0.049 OD	P<0.01
Desmin (2 months)	0.194±0.094 OD	0.489±0.09 OD	0.753±0.053 OD	P<0.01

The data was presented as mean±standard error.

Acetylcholine R referred to acetylcholine receptor.

The abbreviation of control, ES, AFS: see text.

**Fig 5:**
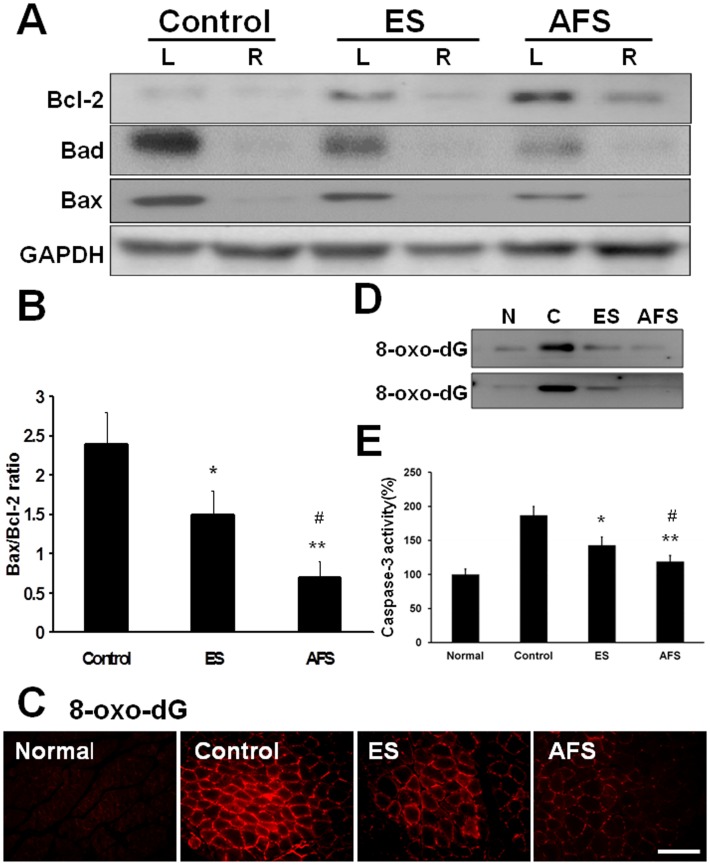
Expression of apoptotic factors in denervated muscle 2 weeks after denervation. (A) Expression of Bcl-2, Bad, and Bax in denervated muscle subjected to different treatment (B) Quantitative analysis of apoptotic markers in different treatment groups (C) The expression of 8-oxo-dG in normal, control, ES, and AFS group. (D) The representative of western blot analysis of 8-oxo-dG related to different treatment (n = 2) (F) Quantitative analysis of caspase-3 in different treatment groups Bar length = 100 μm. R = right, L = left, N = 6 for each group, * p<0.05 and **p<0.01indicated a statistical difference compared to the control group; # p<0.05 indicated a statistical difference as compared to the ES group.

Denervated muscle was subjected to western blot analysis; the significant expression of Bcl-2 was accompanied by a reciprocal decrease of Bad and Bax as noted in the ES and AFS groups (Fig 5A). There was significant reduction of the ratio of Bax/Bcl-2 in the ES group (1.5±0.3) as compared to the control group (2.4±0.4) (p<0.05). A further decrease of this ratio was present in AFS group (0.7±0.2) compared to the control group (p<0.01) or the ES group (p<0.05) (Fig 5B). There were also significant expression of 8-oxo-dG in denervated muscle and decreased its expression when subjected to AFS treatment and electrical stimulation. There was no expression in the normal muscle tissue (Fig 5C). The representative of western blot analysis in 8-oxo-dG in denervated muscle was shown in Fig 5D. The quantitative analysis of caspase-3 activity was illustrated in Fig 5E. The data showed muscle denervation increased the expression of caspase-3 activity and counteracted by ES and further alleviated by AFS. Hence, the administration of AFS in denervated muscle appeared to protect the muscle from apoptosis through the secretion of neurotrophic factors from human AFS.

### Improved neurobehavior after intramuscular injection

Increased nerve regeneration after nerve anastomosis was demonstrated by a decrease in sciatic nerve function index, increased compound muscle action potential, reduced nerve conduction latency, and increased muscle weight. Treatment with human AFS exerted a significant improvement on SFI compared with findings obtained in either the control (p<0.01) or ES groups (p<0.05) (Table 5). The parameters of CAMP, conduction latency, and muscle weight were also restored by human AFS injection as compared to either the control or ES groups (p<0.01, p<0.05, p<0.05, respectively) (Table 6).

**Table 5:** Results of SFI in different groups related for various time frames.

Weeks Groups	1	2	3	4	5	6	7	8
Control	100±1.8	-97.1±1.5	-85.7±1.9	-82.9±1.7	-75.4±1.6	-70.1±1.8	-65.8±2.1	-60.4±1.7
ES	-97.3±1.2	-88.7±1.2	-79.6±1.2	-69.8±1.2	-65.1±1.3	-59.6±1.4	-56.1±1.9	-51.3±1.2
AFS	-94.7±2.6	-84.5±1.5	-74.3±1.4	-65.2±1.3	-61.4±1.4	-55.3±1.2	-46.1±1.8	-41.1±1.1

The p value between the control and ES group was 0.047.

The p value between the control and AFS group was 0.007.

The p value between the AFS and ES group was 0.021.

N = 6 for each group.

Results presented as mean± standard error.

Control, ES, AFS, SFI: see text.

**Table 6:** Outcome of electrophysiology and muscle weight 2 months following the experiment.

	Control	ES	AFS	p-value
Latency	228.1 ± 25.1	179.3±11.3	152.1±11.1	<0.05
CMAP	24.8±3.2	36.1±4.1	51.2±5.1	<0.01
Muscle weight	40.1±2.7	51.4±3.1	66.5±3.7	<0.05

Data presented as percentage of left side divided by right side and mean± standard error.

Control, ES, and AFS: see text.

CMAP: compound muscle action potential.

N = 6 for each group.

**Fig 6:**
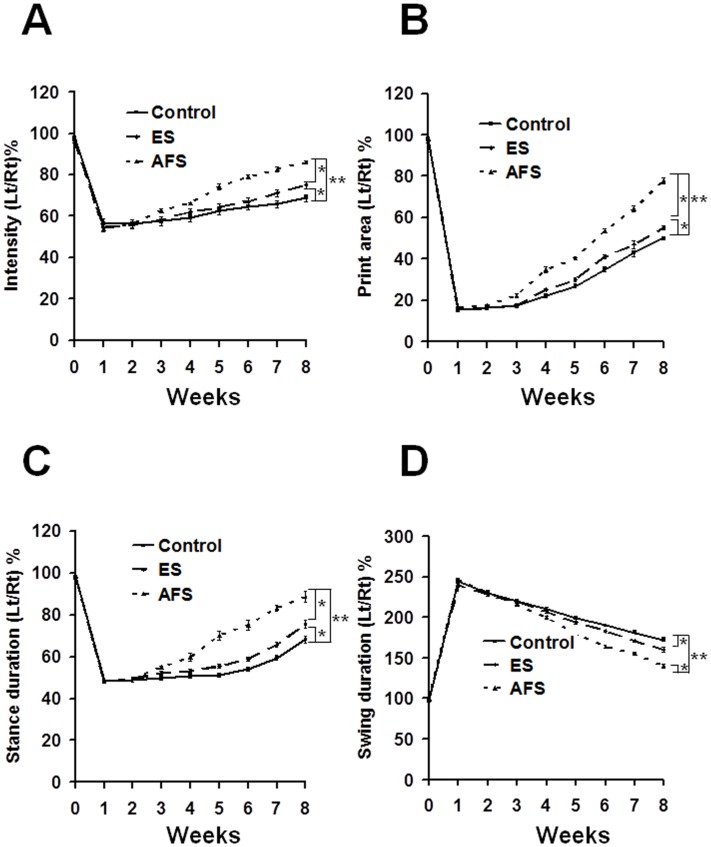
Illustration of various parameters of the CatWalk XT system subjected to various treatments and at different time frames. (A) Illustration of the intensity in different treatment groups (B) Illustration of the print area in different treatment groups (C) Illustration of the stance duration in different treatment groups (D) Illustration of the swing duration in different treatment groups. *p<0.05; **p<0.01 N = 6 for each group.

Static and dynamic functional assessments were performed using the CatWalk system, an automatic analysis for evaluating sciatic nerve injury. The mean intensity and printed area showed significant improvement in the AFS group compared to the control group (p<0.01, p<0.01, respectively) or the ES group (p<0.05, p<0.05, respectively). The ES group showed a significant increase in neurobehavior as compared to the control group (p<0.05, p<0.05, respectively) (Fig 6A and 6B).

In the stance duration, the ratio of duration dropped to half of the original value, and, then, exhibited further improvement in the AFS group as compared to the control (p<0.01) or ES group (p<0.05), respectively. The ES group also showed a significant improvement in stance duration as compared to control group (p<0.05) (Fig 6C). In the swing duration, the ratio of a duration showed a significant decrease in the AFS group compared to the control (p<0.01) or ES groups (p<0.05), respectively. Also, the ratio of duration was significantly decreased in the ES group compared to the control group (p<0.05) (Fig 6D). However, there was no significant difference in step sequence or regularity index between the two groups (data not shown).

### Increased motor neuron survival after intramuscular injection

**Fig 7:**
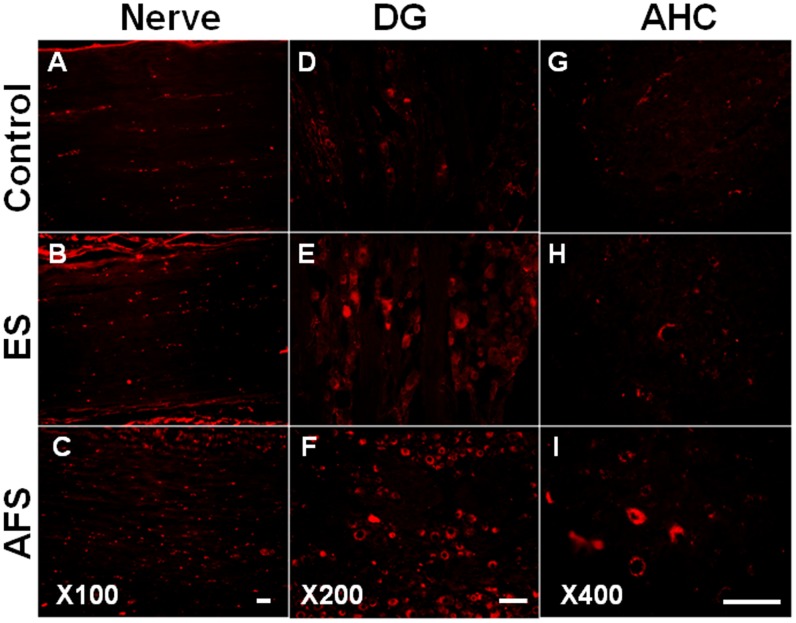
Representative of CTB tracer (Cholera toxin B) distributed to nerve, dorsal root ganglion and anterior horn cells. (A, B, C) Illustration of CTB tracer over the proximal end of nerve in different treatment groups (magnification X 100) (D, E, F) Illustration of CTB tracer over dorsal root ganglion in different treatment groups (magnification X 200) (G, H, I) Illustration of CTB tracer over the anterior horn cells in different treatment groups (magnification X 400). Bar length = 100 μm, n = 6 for each group.

Motor neuron apoptosis was responsible for peripheral nerve injury. Two months after nerve anastomosis, the intra-nerve injection of Cholera toxin B (CTB) showed the significant distribution of CTB over the proximal end of nerve (Fig 7A, 7B and 7C), dorsal root ganglion (Fig 7D, 7E and 7F) as well as anterior horn cells (Fig 7G, 7H and 7I) in AFS and ES group as compared to the control group (p<0.05, p<0.01, p<0.05, respectively). In addition, AFS group also showed a significant increase of CTB expression compared to the control group (Table 7). In neuronal tracking, we found that the intramuscular injection of AFS preserved motor neuron loss as well as dorsal root ganglion cells.

**Table 7:** Results of CTB tracing in different tissues after treatment 2 months after the experiment.

	Control	ES	AFS	p-value
Proximal nerve (Relative density)	21±1.7	34±3.9	57±4.5	p<0.05
Dorsal root ganglion (Number/ mm^2^)	15±2.7	48± 4.3	91.5±5.3	P<0.01
Anterior horn cells (Number/ mm^2^)	2±0.7	12±1.5	25±2.4	P<0.05

Results presented as mean± standard error.

Control, ES, AFS, CTB: see text.

N = 6 for each group.

### Increased regeneration in nerve anastomosis following AFS intramuscular injection

**Fig 8:**
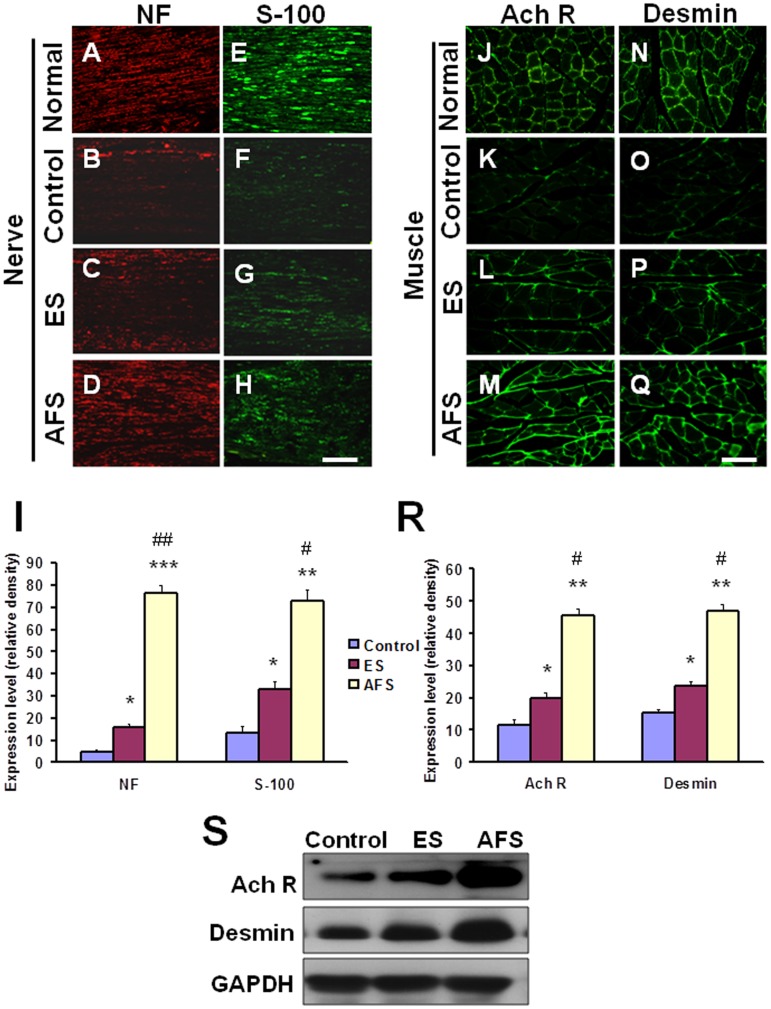
Representative of regeneration in the distal end of the nerve and denervated muscle 2 months after nerve anastomosis. (A, B, C, D) Expression of neurofilament staining in the normal nerve and in the distal end of nerve subjected to different treatments. (E, F, G, H) Expression of S-100 staining in the normal nerve and in the distal end of nerve subjected to different treatments. (J, K, L, M) Expression of acetylcholine receptor staining in normal muscle and the denervated muscle subjected to different treatments. (N, O, P, Q) Expression of desmin staining in the normal muscle and the denervated muscle subjected to different treatments (I) Quantitative analysis of the density of neurofilament and S-100 in distal end of nerve subjected to different treatments (R) Quantitative analysis of the density of acetylcholine receptor and desmin in the denervated muscle subjected to different treatments. (S) The representative of western blot analysis in different treatment related to expression of desmin and Ach R (acetylcholine receptors). Bar length = 100 μm, N = 6 for each group, *p<0.05, **p<0.01 indicated the statistical difference as compared to the control group; # p<0.05, ##p<0.01 indicated the statistical difference as compared to the ES group.

Increased expression of S-100 and neurofilament reflected nerve regeneration response to nerve injury in a long term follow up (Fig 8). The significant expression of neurofilament (Fig 8C and 8D) and S-100 (Fig 8G and 8H) were demonstrated in distal end of the nerve anastomosis in either in the ES or AFS groups as compared to control group (Fig 8B and 8F). High expression of neurofilament and S-100 was demonstrated in normal nerve (Fig 8A and 8E). The AFS exerted a significant increase in neurofilament expression as compared to the ES group (P<0.01, p<0.05, respectively) and control group (p<0.001, p<0.01, respectively) (Fig 8I). High expression of acetylcholine receptor and desmin were shown in normal muscle (Fig 8J and 8N). The density of the acetylcholine receptor in the gastrocnemius muscle was significantly greater in AFS treatment group as compared to the control (p<0.01) (Fig 8M, 8K, 8R and 8N) or ES group (p<0.05) (Fig 8M, 8L and 8R). In the ES group alone, there was a significant increase in the acetylcholine receptor density as compared to control group (p<0.05) (Fig 8L, 8K and 8R) The ES group also showed a significant increase in density of desmin as compared to the control group (p<0.05) (Fig 8P, 8O and 8R). Furthermore, the expression of desmin density in AFS group was greater than that in the ES (p<0.05) (Fig 8Q, 8P and 8R) and the control group (p<0.05) (Fig 8Q, 8O and 8R). The western blot analysis also showed the same trends (Fig 8S). The quantitative analysis by ELISA in Table 6 substantially showed the significant difference in treatment related to the expression of desmin and acetylcholine receptors.

**Fig 9:**
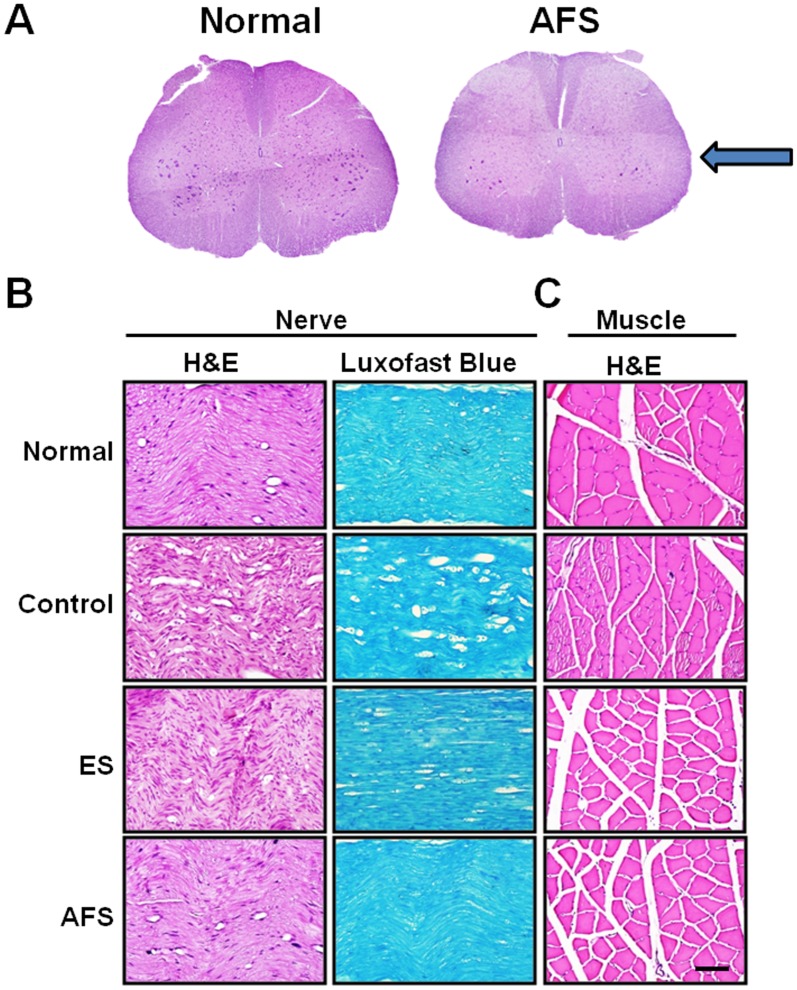
Illustration of morphology alteration of spinal cord, nerve, and muscle 2 months after nerve anastomosis. (A) morphology alteration in anterior horn cells subjected to AFS treatment (B) morphology and myelination of the distal end of nerve subjected to treatment group (C) morphology alteration in denervated muscle subjected to different treatment group. Bar length = 100 μm; arrow indicated the site of nerve injury.

In histology analysis, we found that the number of anterior horn cells was restored by AFS treatment and approached the normal spinal cord (Fig 9A). The structure of nerve and myelination was also maintained by AFS and ES. In AFS treatment, the morphology was very similar to the normal nerve (Fig 9B). The size of muscle bundle was decreased subjected to nerve injury. The size of muscle bundle was escalated by ES and furthermore by AFS treatment. The result of AFS treatment approached the one in normal muscle tissue (Fig 9C). Based on the abovementioned findings, the intramuscular injection of AFS promoted significantly increased regeneration of nerve and muscle tissue function as compared to the control group.

## Discussion

Intramuscular injection of human AFS rescued the muscle from apoptosis, and, furthermore, it supported nerve regeneration mainly through the secretion of neurotrophic factors from human AFS. In the long term analysis, we found that the intramuscular injection of AFS can prevent the muscle from denervation, and this effect increases nerve regeneration and promotes motor neuron and dorsal root ganglion survival. Thus, intramuscular injection may be an alternative treatment option in nerve injury either short term for reducing denervation injury or long term for facilitating nerve regeneration.

Other studies have shown that AFS cells have the potential to secrete several neurotrophic factors to assist the muscle and nerve regeneration [21; 26–31]. In our previous study, AFS transplantation promoted nerve regeneration, possibly through the secretion of CNTF and NT-3 [26,27]. In addition, AFS cells transplantation enhanced GDNF secretion and escalated the modulation of the inflammatory process [28–30]. Intravenous administration of human AFS improved nerve and muscle regeneration was highly correlated with survival of AFS cells and associated with expression of CNTF, NT-3 and BDNF [31]. In this study, we found that high expression of neurotrophic factors such as NT-3, BDNF, CNTF, and GDNF was present in human AFS. Intramuscular injection of AFS not only caused a significant increase of neurotrophic factors in muscle, but it also augmented neurotrophic factors distribution in the distal end of the injured nerve. This result was compatible with previous reports [26,27]. Thus, this inherent neurotrophic factors secretion from AFS supported the survival of denervated muscle and, furthermore, increased the nerve regeneration.

Apoptosis is an organized dismantling of cells with defining morphologic features that include plasma membrane blebbing, nuclear breakdown, and DNA fragmentation. Mitochondria are involved in apoptosis because they contain pro-apoptotic molecules, which can be released to initiate cell death. Mitochondria are also the primary cellular producer of reactive oxygen species [11]. Muscle denervation can induce changes in mitochondrial contents that result in an elevation of Bax and a reduction of Bcl-2 as well as a significant rise in the ratio of Bax to Bcl-2 [11]. In this study, denervation caused a significant increase in the ratio of Bax to Bcl-2. However, intramuscular injection of AFS rescued muscle from apoptosis in part through a decrease in the ratio of Bax to Bcl-2. This reduction in apoptosis was consistent with the increased acetylcholine receptor expression and preservation of muscle morphology both of which are hallmarks of muscle regeneration [32–35].

The expression of acetylcholine receptor at the neuromuscular junction is a typical feature of regeneration of a denervated muscle [32,33]. The up-regulation of acetylcholine receptors in animals treated with intravenous injection of AFS is compatible with an increased number of axon myelination as well as improved neurological function [31]. In addition, desmin is also important in muscle cell architecture and structure since it connects many components of cytoplasm and forms a scaffold around the Z disc of the sarcomere [34]. It also connects the contractile apparatus of the cell nucleus, mitochondria and post-synaptic area of motor endplates and maintains the structural and mechanical integrity of muscle cells during the contraction [35]. Intramuscular injection of AFS increased the expression of the acetylcholine receptor and desmin either in the short term during muscle denervation or the long term during nerve anastomosis. These effects further confirmed that intramuscular injection of AFS could rescue the denervated muscle from apoptosis and augment the improvement of neurological outcome.

Based on the current experiments, we found that the expression of neurotrophic factors in AFS delivered to denervated muscle rescued the denervated muscle from apoptosis as well as supported nerve regeneration in the long-term. In order to further confirmation this hypothesis, a knock-down of neurotrophic factors by si NRA should be conducted in a future study.

## Conclusion

Intramuscular injection of human AFS may attenuate apoptotic denervation of muscle tissues. This effect was mainly through the secretion of neurotrophic factors from transplanted AFS. The neurotrophic factors not only rescued the muscle atrophy but also promoted nerve remyelination. In a long-term follow up, intramuscular injection of AFS facilitated the muscle and nerve regeneration. Thus, intramuscular injection of AFS could be a potential treatment strategy for nerve repair.
